# Immediate prepectoral versus submuscular breast reconstruction in nipple-sparing mastectomy: a retrospective cohort analysis

**DOI:** 10.61622/rbgo/2024rbgo76

**Published:** 2024-09-06

**Authors:** Francisco Pimentel Cavalcante, Ticiane Oliveira Lima, Ryane Alcantara, Amanda Cardoso, Guilherme Novita, Felipe Zerwes, Eduardo Millen

**Affiliations:** 1 Hospital Geral de Fortaleza Fortaleza CE Brazil Hospital Geral de Fortaleza, Fortaleza, CE, Brazil.; 2 Instituto Oncoclínicas São Paulo SP Brazil Instituto Oncoclínicas, São Paulo, SP, Brazil.; 3 Pontifícia Universidade Católica do Rio Grande do Sul Porto Alegre RS Brazil Pontifícia Universidade Católica do Rio Grande do Sul, Porto Alegre, RS, Brazil.; 4 Clinica São Vicente Rio de Janeiro RJ Brazil Clinica São Vicente, Rio de Janeiro, RJ, Brazil

**Keywords:** Pectoralis muscles, Nipples, Breast implantation, Breast implants, Breast neoplasms, Mammaplasty, Mastectomy, Surgical procedures, operative

## Abstract

**Objective:**

To evaluate early complications in prepectoral breast reconstruction.

**Methods:**

A retrospective cohort study including 180 consecutive cases of nipple-sparing mastectomy, comparing immediate breast reconstruction with subpectoral to prepectoral mammary implants in 2012-2022. Clinical and demographic characteristics and complications in the first three months following surgery were compared between the two techniques.

**Results:**

The prepectoral technique was used in 22 cases (12.2%) and the subpectoral in 158 (87.8%). Median age was higher in the prepectoral group (47 versus 43.8 years; p=0.038), as was body mass index (25.1 versus 23.8; p=0.002) and implant volume (447.5 versus 409 cc; p=0.001). The prepectoral technique was more associated with an inframammary fold (IMF) incision (19 cases, 86.4% versus 85, 53.8%) than with periareolar incisions (3 cases, 13.6% versus 73, 46.2%); (p=0.004). All cases in the prepectoral group underwent direct-to-implant reconstruction compared to 54 cases (34.2%) in the subpectoral group. Thirty-eight complications were recorded: 36 (22.8%) in the subpectoral group and 2 (9.1%) in the prepectoral group (p=0.24). Necrosis of the nipple-areola complex/skin flap occurred in 27 patients (17.1%) in the subpectoral group (prepectoral group: no cases; p=0.04). The groups were comparable regarding dehiscence, seroma, infection, and hematoma. Reconstruction failed in one case per group (p=0.230). In the multivariate analysis, IMF incision was associated with the prepectoral group (aOR: 34.72; 95%CI: 2.84-424.63).

**Conclusion:**

The incidence of early complications was comparable between the two techniques and compatible with previous reports. The clinical and demographic characteristics differed between the techniques. Randomized clinical trials are required.

## Introduction

Oncoplastic breast reconstruction represented an important advance in the treatment of early-stage breast cancer both in breast-conserving surgery and in total mastectomy.^1,2^ The so-called skin-sparing mastectomy (SSM) and nipple-sparing mastectomy (NSM) started to become more popular options, particularly because these techniques facilitate immediate breast reconstruction following maximum preservation of breast skin and the nipple-areola complex (NAC), respectively. Both techniques are used for prophylactic or therapeutic reasons, with adequate oncological local control.^3-5^

Immediate implant-based breast reconstruction, either with two-stage tissue expander or single-stage direct-to-implant (DTI) reconstruction, has increased in popularity in various countries compared to the use of autologous tissue.^6-8^ Traditionally, subpectoral implant placement has been used in such cases, since this submuscular technique provides greater implant coverage and the impact in terms of immediate mastectomy-related complications was less than with subcutaneous implantation.^9,10^ Recent improvements in surgical techniques and better understanding of the risk factors associated with complications have led to the resurgence of prepectoral placement, with or without the use of an acellular dermal matrix (ADM). However, although the prepectoral space lies in the subcutaneous plane, the current prepectoral approach differs considerably from the earliest subcutaneous reconstruction techniques. This present-day technique has the advantages of involving a shorter duration of surgery, less postoperative pain, absence of breast animation deformity and acceptable cosmetic results. Nevertheless, there are no randomized studies on this scenario, but retrospective reports without follow-up of long-term complications.^11-15^

The principal objective of the present study was to evaluate the clinical and demographic characteristics and early complications in cases of breast reconstruction, comparing the prepectoral technique with the subpectoral technique in patients submitted to NSM in a single institution.

## Methods

This retrospective cohort study was designed to compare the clinical and demographic characteristics and the complications arising in the first three months after immediate breast reconstruction using the prepectoral or subpectoral technique. In all cases, the patients had undergone NSM, for either therapeutic or prophylactic reasons, and implant-based reconstruction (single-stage DTI or two-stage tissue expander reconstruction) between 2012 and 2022. The same surgical team operated on all the patients. The data evaluated were collected from the patient records. The exclusion criteria consisted of having undergone chest wall radiotherapy prior to surgery; any type of mastectomy other than NSM; breast reconstruction using myocutaneous flaps; failing to undergo immediate breast reconstruction; and incomplete medical records.

The following clinical and demographic characteristics of the patients were collected, analyzed and compared between the groups: mean age (also dichotomized into >45 years or <45 years); mean volume of the implant or tissue expander (also dichotomized into >350 cc or <350 cc); the presence of comorbidities (hypertension, diabetes mellitus, autoimmune diseases); current smoking; mean body mass index (BMI), (also categorized into ≤25, >25-30, >30); reason for surgery (therapeutic or prophylactic); and the type of incision performed. Early postoperative complications were defined as any degree of necrosis of the NAC or of the skin flap, dehiscence above 5 mm, the presence of seroma requiring aspiration, infection, hematoma, and failed reconstruction with loss of the implant or tissue expander.

In all cases, before administering general anesthesia, with the patient in the seated position, skin marking was performed and the margins of the area to be resected were outlined. The anatomical planes of the skin flap were dissected using an electric scalpel, up to the projection of the previously marked area of the skin, preserving the fascia of the pectoralis major and the subcutaneous layer of the breast by dissecting at the superficial fascia. In prepectoral reconstruction, the implant or tissue expander was inserted directly above the pectoralis major, without the use of an ADM or surgical mesh (these products are unavailable at this institute). For the subpectoral technique, the pectoralis major was previously dissected and partially detached from the chest wall using an electric scalpel, creating a partial or complete subpectoral pocket, with or without the serratus anterior (or its fascia), where the implant or tissue expander was placed. In cases in which the axillary approach was used, a separate incision in the axilla was generally made in patients submitted to the inframammary fold (IMF) incision. Drains were always placed in a lateral position to the reconstructed breast and were preferentially removed when the drain debit was less than 30 ml in 24 hours. Antibiotics were maintained until the drains were removed. In general, the patients were monitored regularly with weekly clinical examinations in the first month and then monthly for the next two months or at the criteria of the surgical team in case of complications. The treatment of early-stage breast cancer followed the international guidelines for neoadjuvant and adjuvant therapy. Likewise, international recommendations were used regarding the follow-up provided after adjuvant treatment was complete.

The data were tabulated using Microsoft Excel and then exported to SPSS, version 20.0 for Windows. Analyses were performed with the confidence level set at 95%. The continuous variables were expressed as means, medians, standard deviations, and maximum and minimum values, and the categorical variables as frequencies and percentages. Student’s t-test was used to compare the means of the quantitative variables between the types of incision, while Fisher’s exact test or Pearson’s chi-square test was applied to compare the qualitative variables between the groups, including the complications. Finally, clinical and demographic variables that were significantly associated with complications in the univariate analysis were included in the multivariate analysis. P-values <0.05 were considered statistically significant throughout the analysis.

The study was conducted according to current ethical regulations and in compliance with the Declaration of Helsinki. The institutional internal review board approved the study protocol prior to commencement under reference number 5.185.247 (CAAE: 53372921.9.0000.5040). This article was prepared in accordance with the STROBE statement for observational studies.

## Results

Overall, 180 cases of NSM associated with immediate breast reconstruction performed between 2012 and 2022 were included. Of these, 22 breast reconstructions (12.2%) were performed using the prepectoral technique and 158 (87.8%) with the subpectoral technique. The mean age of patients was 47 years and 43.8 years in the prepectoral and subpectoral groups, respectively (p=0.038). Ages ranged from 27 to 74 years and when age was dichotomized into younger or older than 45 years of age, no statistically significant difference was found between the groups (p=0.65). The mean volume of the implant or tissue expander was greater in the prepectoral group (447.5 cc versus 409 cc in the subpectoral group; p=0.001); however, when evaluated according to whether the volume used was greater or less than 350 cc, no statistically significant difference was found between the groups (p=0.46). There was also no difference in terms of comorbidities, with 3 patients (13.6%) having comorbidities in the prepectoral group compared to 20 (12.7%) in the subpectoral group (p=0.69). There was also no difference between the groups in terms of the reason for surgery, with 13 cases (59.1%) being therapeutic and 9 (40.9%) prophylactic in the prepectoral group compared to 74 cases (46.8%) of therapeutic mastectomy and 84 (53.2%) cases of prophylactic surgery in the subpectoral group (p=0.36). The BMI of patients in the prepectoral group was higher (25.1 versus 23.8; p=0.002), since 15 (68.2%) patients in the prepectoral group were overweight compared to 43 (27.2%) in the subpectoral group (p=0.0005). The patients in the prepectoral group were more likely to have had an IMF incision: 19 (86.4%) versus 85 (53.8%), with fewer having a periareolar incision: 3 (13.6%) versus 73 (46.2%) (p=0.004). All the prepectoral breast reconstructions consisted of DTI reconstructions, while in the subpectoral group DTI was used in 54 cases (34.2%) (Table 1).

**Table 1:** Clinical and demographic characteristics of the patients submitted to prepectoral or subpectoral breast reconstruction

Variables	Total n(%)	Surgical technique		p-value
		Prepectoral n(%)	Subpectoral n(%)	
Age (years)	45.18 ± 10.08	47.00 ± 10.12	43.86 ± 9.89	0.038 *
BMI	24.41 ± 2.70	25.13 ± 2.95	23.88 ± 2.38	0.002 *
Implant volume (cc)	425.28 ± 77.85	447.50 ± 76.05	409.04 ± 75.44	0.001 *
Comorbidities				
No	157(87.2)	19(86.4)	138(87.3)	0.694
Yes	23(12.8)	3(13.6)	20(12.7)	
Reason for surgery				
Cancer	87(48.3)	13(59.1)	74(46.8)	0.3635
Prophylactic	93(51.7)	9(40.9)	84(53.2)	
Type of implant				
Implant (DTI)	76(43.2)	22(100.0)	54(34.2)	7.535e-10*
Tissue expander	104(56.8)	0(0.0)	104(65.8)	
Type of incision				
IMF	104(57.8)	19(86.4)	85(53.8)	0.004746*
Periareolar	76(42.2)	3(13.6)	73(46.2)	

BMI - body mass index; DTI - direct-to-implant; IMF - inframammary fold. *p<0.05 Fisher’s exact test, Pearson’s chi-square test or Student’s t-test (mean ± standard deviation)

A total of 38 complications were recorded: 36 (22.8%) in the subpectoral group and 2 (9.1%) in the prepectoral group (p=0.24) (Table 2). When evaluated by type of breast reconstructive surgery, there was no difference between complications using a subpectoral tissue expander (14/54; 25.9%) or subpectoral implant (22/104; 21.1%) (p=0.4975), as well as when both are compared to prepectoral implant reconstruction (2/22; 9.1%) (p=0.2644).

**Table 2:** Overall incidence of complications according to the use of the prepectoral or subpectoral reconstruction technique

Complications	Total n(%)	Surgical technique		p-value
		Prepectoral n(%)	Subpectoral n(%)	
No	142(78.9)	20(90.9)	122(77.2)	
Yes	38(21.1)	2(9.1)	36(22.8)	0.2436

*p<0.05 Fisher’s exact test or Pearson’s chi-square test

Necrosis of the NAC or skin flap occurred in 27 cases in the subpectoral group (17.1%), with no such cases being recorded in the prepectoral group (p=0.04). There was no statistically significant difference in relation to any of the other complications: dehiscence: 1 (4.5%) in the prepectoral group and 4 (2.5%) in the subpectoral group, (p=0.483); seroma: 1 (4.5%) versus 5 (3.2%), (p=0.548); infection 0 versus 3 (1.9%), (p=1); and hematoma 0 versus 1 case (0.6%), (p=1). Breast reconstruction failed in two cases, one in the prepectoral group and the other in the subpectoral group, (p=0.230) (Table 3).

**Table 3:** Incidence of complications according to type and the use of the prepectoral or subpectoral reconstruction technique

	Total n(%)	Surgical technique		p-value
		Prepectoral n(%)	Subpectoral n(%)	
Necrosis				
No	153(85.0)	22(100.0)	131(82.9)	0.04751*
Yes	27(15.0)	0(0.0)	27(17.1)	
Dehiscence				
No	175(97.2)	21(95.5)	154(97.5)	0.483
Yes	5(2.8)	1(4.5)	4(2.5)	
Seroma				
No	174(96.7)	21(95.5)	153(96.8)	0.548
Yes	6(3.3)	1(4.5)	5(3.2)	
Infection				
No	177(98.3)	22(100.0)	155(98.1)	1
Yes	3(1.7)	0(0.0)	3(1.9)	
Hematoma				
No	179(99.4)	22(100.0)	157(99.4)	1
Yes	1(0.6)	0(0.0)	1(0.6)	
Failed breast reconstruction				
No	178(98.9)	21(95.5)	157(99.4)	0.2301
Yes	2(1.1)	1(4.5)	1(0.6)	

*p<0.05 Fisher’s exact test or Pearson’s chi-square test

In the multivariate analysis, none of the clinical or demographic factors evaluated were associated with complications. IMF incisions were associated with the prepectoral group (adjusted odds ratio: 34.72; 95%CI: 2.84 - 424.63) (Table 4).

**Table 4:** Multivariate analysis to evaluate the association of clinical and demographic characteristics with complications

Clinical and demographic characteristics	p-value	Adjusted OR (95%CI)
Age	0.060	-
Implant volume	0.618	-
Body mass index	0.995	-
Comorbidities	0.992	-
Reason for surgery	0.288	-
Type of implant	0.938	-
Type of incision	0.005^*^	34.72 (2.84-424.63)

*p<0.05, multinomial logistic regression. OR: odds ratio; 95%CI: 95% confidence interval

## Discussion

Immediate implant-based breast reconstruction represented a major step forward in the surgical treatment of breast cancer, improving the quality of life of women submitted to mastectomy, while maintaining acceptable results over the long term and in many cases avoiding the complications associated with techniques that use myocutaneous flaps. The subpectoral technique was traditionally used in this type of reconstruction, minimizing the rate of complications over the years and facilitating the dissemination of immediate breast reconstruction.^16^ Over recent years, however, understanding on the physiopathology involved in implant-related complications has increased. The risk of bacterial biofilm formation and the improvement in surgical techniques, including the advent of conservative mastectomy techniques such as NSM and SSM, have resulted in less radical oncologic resections, giving rise to a greater interest in prepectoral implant-based breast reconstruction. This technique introduces benefits associated with the non-use of muscle coverage, including a shorter surgery time for breast reconstruction, less postsurgical pain and avoidance of animation deformity in the reconstructed breast.^2,3,11^ The advent of adjuvant and neoadjuvant systemic therapy could also be considered an important factor in the resurgence of prepectoral techniques since these treatments decreased the rates of local breast cancer recurrence and ultimately allowed the extent of surgery to be reduced.^17^ Indeed, more radical surgeries are generally associated with a higher complication rate in immediate breast reconstruction. In a study involving 1,212 cases of NSM, 40% therapeutic and 60% prophylactic, the therapeutic cases were associated with more complications, including infection (p=0.04), seroma (p=0.04) and failed breast reconstruction (p=0.005).^18^ In the present study, no statistically significant differences were found between prophylactic and therapeutic surgeries with regards to complications, possibly reflecting these recent advances in surgery and in the systemic treatment in this practice; however, some differences were found in the clinical and demographic profile.

As with any new surgical technique, it is crucial to continue evaluating complications to improve understanding, including the assessment of complications occurring over the long term. However, this was not one of the objectives of the present study, in which data were collected for up to three months following surgery. Various series have reported acceptable early complication rates with the prepectoral compared to the subpectoral position. A meta-analysis of 13 studies that included a total of 4,692 breasts in 3,014 patients showed similar rates of infection (odds ratio [OR] 0.74; 0.556-1103), wound dehiscence (OR: 0.947; 0.555-1.618), seroma (OR: 1125; 0.773-1637), hematoma (OR: 1.592; 0.958-2.645) and loss of the implant (OR: 0.895; 0.602-1226) between the prepectoral and subpectoral groups; however, the rate of necrosis was higher with subpectoral reconstruction (OR: 0.665; 0.464-0.952).^11^ These data are compatible with the results of the present study, which showed similar overall early complication rates with the prepectoral and subpectoral techniques but a higher rate of necrosis (of the skin flap and NAC) in the subpectoral group. Nevertheless, the findings reported here could be linked to the higher rate of periareolar incision used in this group, which is generally known to result in a higher rate of necrosis, particularly of the NAC. Indeed, a recent meta-analysis of 9,975 cases of NSM showed a higher rate of NAC necrosis with the periareolar incision (18%) compared to other types of incision, with IMF incisions being one of those with the lowest rate of NAC necrosis.^19^

Patients submitted to the prepectoral technique in this study had a higher BMI compared to those in the subpectoral group. This finding is unsurprising, since reconstruction is theoretically simpler with the prepectoral technique when the subcutaneous tissue is thicker. Furthermore, a recent retrospective analysis reported a lower complication rate in 133 patients with BMI >30 submitted to prepectoral compared to subpectoral reconstruction, including seroma (p=0.003) and infection (p=0.018).^20^ Overweight could also, theoretically, minimize the impact of rippling over the long term due to the increased thickness of the subcutaneous tissue. However, this factor was not evaluated in the present study. Indeed, some studies have shown that rippling could be an important concern in the prepectoral technique and has been reported in up to 35% of cases.^21^ Although radiotherapy is increasingly used as part of the current arsenal of breast cancer treatment, its impact on immediate prepectoral reconstruction was not evaluated in the present study.^17^ Some analyses, however, have reported acceptable complication rates following adjuvant radiotherapy, including rates of capsular contracture. In a meta-analysis of 21 studies that included cases in which the prepectoral technique and radiotherapy were used (n=423), capsular contraction was lower with the prepectoral technique compared to subpectoral implant-based breast reconstruction (OR: 0.480; 0.285-0.808).^11^

Another important finding of the present study was that all the patients in the prepectoral group underwent DTI breast reconstruction, another trend observed in recent years that can reduce the costs to the hospital and the patient in appropriate cases.^22^ This combination of techniques seems interesting, even without the use of ADM or meshes. Meshes are not used in this institute, as they are not available. However, in the majority of cases reported, these tools failed to have any significant impact in immediate implant-based breast reconstruction.^23^ A recent randomized study compared immediate implant-based breast reconstruction, with or without ADM, in 135 women operated on between 2014 and 2017 and reported no statistically significant difference between the groups with respect to repeat surgery or cosmetic outcome.^24^

There are certain limitations associated with the present study, therefore these results must be evaluated with caution. First, this was a retrospective series involving a small number of cases of prepectoral breast reconstruction and a short follow-up time. Consequently, important factors such as the impact of radiotherapy on prepectoral breast reconstruction and the incidence of rippling over the long term were not evaluated. Furthermore, prepectoral reconstruction may have been recommended in cases considered “ideal” for the technique; hence, with the aim of minimizing postsurgical complications. Therefore, conclusions cannot be made regarding which of the techniques is better insofar as early complications are concerned. Indeed, the objective of this study was to describe the profile of patients undergoing these initial techniques and to evaluate the principal complications associated with each technique. On the other hand, these data present new perspectives for this type of breast reconstruction, which, when taken together with the results of other studies, allow the conclusion to be reached that mastectomy with immediate implant-based breast reconstruction is possible in many cases within a conservative approach, both skin- and muscle-sparing. Nonetheless, more data are required on this surgical technique, preferably from randomized clinical trials.^25^

## Conclusion

The clinical and demographic characteristics associated with prepectoral and submuscular breast reconstruction differed between the two techniques. The overall complication rates with prepectoral breast reconstruction following nipple-sparing mastectomy were comparable to those found with the subpectoral technique and compatible with rates previously reported in the literature. More data are required, particularly from randomized clinical trials, to confirm these findings.
